# Short-Term Angiotensin II Treatment Affects Large Artery Biomechanics and Function in the Absence of Small Artery Alterations in Mice

**DOI:** 10.3389/fphys.2018.00582

**Published:** 2018-05-16

**Authors:** Arthur J. A. Leloup, Sofie De Moudt, Cor E. Van Hove, Lindsey Dugaucquier, Zarha Vermeulen, Vincent F. M. Segers, Gilles W. De Keulenaer, Paul Fransen

**Affiliations:** ^1^Laboratory of Physiopharmacology, Department of Pharmaceutical Sciences, University of Antwerp, Antwerp, Belgium; ^2^Laboratory of Pharmacology, Faculty of Medicine and Health Sciences, University of Antwerp, Antwerp, Belgium; ^3^Department of Cardiology, Antwerp University Hospital, Edegem, Belgium; ^4^Department of Cardiology, Middelheim Hospital, Antwerp, Belgium

**Keywords:** angiotensin II, mouse aorta, arterial stiffness, basal NO, vascular smooth muscle cell

## Abstract

Induction of hypertension by angiotensin II (AngII) is a widely used experimental stimulus to study vascular aging in mice. It is associated with large artery stiffness, a hallmark of arterial aging and a root cause of increased cardiovascular risk. We reported earlier that long term (4 week) AngII treatment in mice altered the active, contractile properties of the arteries in a vascular bed-specific manner and that, in healthy mice aorta, active contractile properties of the aortic wall determine isobaric aortic stiffness. Given the huge physiological relevance of large artery stiffening, we aimed to characterize the early (1 week) changes in the active properties of the aorta of AngII-treated mice. We were not able to detect a significant effect of AngII treatment on anesthetized blood pressure or abdominal aorta pulse wave velocity. *Ex vivo* biomechanical and functional studies of the aorta revealed increased arterial stiffness and altered vascular smooth muscle cell (VSMC) and endothelial cell reactivity. Interestingly, the AngII-associated changes in the aorta could be largely attributed to alterations in basal VSMC tone and basal nitric oxide efficacy, indicating that, besides structural remodeling of the arterial wall, dysfunctional active components of the aorta play a crucial role in the pathophysiological mechanisms by which AngII treatment induces arterial stiffness.

## Introduction

Progressive large artery stiffening is the predominant cause of increased pulse pressure, a marker of cardiovascular (CV) risk in the general population (Benetos et al., 1997) and a predictor of CV events (Mitchell et al., 1997). It reduces myocardial perfusion efficiency, increases left ventricular afterload and elicits mechanical stress on capillaries, potentially damaging the capillary wall of strongly perfused organs such as the heart, brain and kidneys (Safar et al., 2012). It is generally assumed that arterial stiffness is an adaptation mechanism of the aortic wall to increased distending pressures. However, the REASON study, published in 2009, states that, in hypertensive patients, high arterial stiffness predicts a poor response to antihypertensive treatment (Protogerou et al., 2009). In addition, several epidemiological studies report that arterial stiffness precedes hypertension in the elderly (Najjar et al., 2008; Kaess et al., 2012). This temporal relationship was recently confirmed in a mouse model of diet-induced obesity; i.e., arterial stiffness preceded hypertension and target organ damage in this model (Weisbrod et al., 2013). These observations suggest an etiological role for arterial stiffness in the pathogenesis of vascular aging and, hence, a potential therapeutic target to treat CV disease.

The mechanisms of arterial stiffening are complex and incompletely understood. In recent years, there is emerging evidence that not only passive alterations such as elastin fiber degradation determine arterial compliance. Indeed, vascular smooth muscle cell (VSMC) stiffness and active vessel wall components [i.e., nitric oxide (NO) bioavailability and VSMC tonus] affect arterial compliance as well (Fitch et al., 2001; Kinlay et al., 2001; Bellien et al., 2010; Sehgel et al., 2013). We recently developed a novel set-up to isobarically measure biomechanical properties of the isolated mouse aorta and revealed a remarkably high contribution of VSMC tone and basal NO efficacy to the isobaric biomechanical properties of isolated aortic segments of healthy mice (Leloup et al., 2016). Although the interest in the role of active components (i.e., VSMC and EC cross-talk) as potential therapeutic targets has grown, their definite role in arterial stiffening during disease processes is still incompletely understood, increasing the need for further research on the role of active vessel wall components in arterial stiffening.

Angiotensin II (AngII) is a peptide hormone of the renin-angiotensin-aldosterone system and elicits pleiotropic effects on the vasculature. It is a potent vasoconstrictor and acts via the AngII receptor 1 (AT1) that is predominantly expressed on resistance vessels, hence AngII increases blood pressure through increasing peripheral resistance. In the thoracic mouse aorta, the expression of the contractile AT1b receptor is low and aortic rings do not contract upon AngII stimulation *ex vivo* (Russell and Watts, 2000; Zhou et al., 2003; Fransen et al., 2016). However, we previously showed that 4-week AngII treatment in mice does alter the active properties of the VSMCs and ECs in a vascular bed-specific manner. In the aorta, it significantly sensitized isometric contractions induced by elevated extracellular K^+^ and depolarization. Consistent with these observations, AngII-treatment also sensitized the vaso-relaxing effects of the L-type Ca^2+^ channel blocker diltiazem during K^+^-induced contractions. On the other hand, AngII-treatment desensitized the isometric contractions to depolarization in femoral arteries (Fransen et al., 2016). AngII can – independently from its vasoconstrictor effect on resistance arteries – also stimulate inflammation and aortic wall remodeling via the production of reactive oxygen species (ROS), activation of NADPH oxidases and by production of TGF-β (Wang et al., 2001). These combined effects result in hypertrophy of ECs and VSMCs (phenotypic switching), collagen deposition (MMP2 and MMP9 upregulation), infiltration of activated macrophages, expression of VCAM-1, thereby altering the biomechanical properties of the arteries (Fry et al., 2015). Altogether, increased AngII signaling is considered a strong stimulus of arterial aging and AngII treatment in mice affects the structural, molecular, functional and biomechanical properties of the arterial wall (Wang et al., 2001; Majeed et al., 2014; Eberson et al., 2015; Xie et al., 2015; Fransen et al., 2016).

Given the severe, aorta-specific changes in VSMC and EC cross-talk in chronically treated AngII mice (Fransen et al., 2016) and the remarkably large contribution of VSMC tone and basal NO efficacy to isobaric aortic wall stiffness *ex vivo* (Leloup et al., 2016), we aimed to characterize the early (1 week) changes in the aortic wall of AngII-treated mice to assess whether alterations in the active properties of the large arteries contribute to the pathogenesis of arterial stiffening in this widely used mouse model of arterial aging.

## Materials and Methods

### Animals

All animals [male C57BL/6JRj mice, *n* = 20 (Janvier Labs)] arrived in the animal facility of the University of Antwerp at the age of 6 weeks. They were housed in standard cages with 12–12h light-dark cycles with free access to regular chow and tap water. All experiments were performed at the age of 5–6 months. The studies were approved by the Ethical Committee of the University of Antwerp, and all experiments were performed conform to the *Guide for the Care and Use of Laboratory Animals* published by the US National Institutes of Health (NIH Publication No. 85–23, revised 1996).

### AngII Treatment

Mice were randomly divided into groups and treated with saline (*n* = 10) or with AngII (*n* = 10) via subcutaneous osmotic minipumps (model 1007D, Alzet, Cupertino, CA, United States) for 7 days (1000 ng.kg^-1^.min^-1^). Mice were anesthetized using sevoflurane (4–5% in O_2_, 1 l min^-1^). Using a hemostat, a subcutaneous pocket wide enough for an osmotic minipump was created. The pocket was flushed with saline and the osmotic minipump was inserted. After closing the incision with sterile sutures, a single dose of 7.5 mg kg^-1^ ketorolac tromethamine (Ketalar^®^, Pfizer, United States) was subcutaneously administered. Calculations of the required AngII concentrations were made per individual mouse based on its weight and using the online tool provided by Alzet.

### Non-invasive Abdominal Aorta Pulse Wave Velocity (aaPWV) Measurements

High frequency Ultrasound (Vevo 2100, VisualSonics) was used to assess PWV, an important *in vivo* parameter of arterial stiffness, in the abdominal aorta of spontaneously breathing mice under light anesthesia (1.5% isoflurane in 1 l min^-1^ O_2_). Body temperature was maintained at 36–38°C and heart rate at 500 ± 50 beats/min. PWV measurements were performed with a 24-MHz transducer using the method developed by Di Lascio et al. (2014). In short, a 24-MHz transducer was positioned abdominally. 700 frames-per-second B-mode images of the abdominal aorta were obtained using the EKV imaging mode (VisualSonics) to measure aortic diameter (D). A pulse wave Doppler tracing was obtained to measure aortic flow velocity (V). Velocity was plotted against the natural logarithm of the diameter, and the slope of the linear part of the resulting ln(D)-V loop was used to calculate PWV using Matlab v2014 (Mathworks).

### Invasive BP Measurements

Mice were anesthetized using isoflurane (1.5% in 1 l min^-1^ O_2_) and placed supine under a heating lamp. After removal of a distal segment of the clamped right femoral artery (wire myograph experiments, see below), the right carotid artery was cannulated with a 1.4F pressure conductance catheter (Millar), which was advanced through the carotid artery in the aortic arch. Pressure signals were continuously acquired using a data acquisition system (Powerlab 8/30 and LabChart 7, ADInstruments). Stable recordings of arterial pressure pulses were acquired at 1 kHz sampling rate at a heart rate between 400 and 600 bpm. Mice were subsequently euthanized by exsanguination during blood collection from the right carotid artery, under full anesthesia. Data were analyzed using the PV Loop 2.0 module in LabChart. 20-50 stable pressure pulses were averaged for each analysis.

### Wire Myograph

The right femoral and left carotid artery of the isoflurane-anesthetized mice (1.5% in 1 l min^-1^ O_2_) were removed and stripped of adherent tissue. 2 mm segments were mounted in the wire myograph (DMT, Aarhus Denmark) in Krebs Ringer (KR) solution (37°C, 95% O_2_/5% CO_2_, pH 7.4) with (in mM): NaCl 118, KCl 4.7, CaCl_2_ 2.5, KH_2_PO_4_ 1.2, MgSO_4_ 1.2, NaHCO_3_ 25, CaEDTA 0.025, and glucose 11.1. The solution was continuously aerated with a 95% O_2_/5% CO_2_ gas mixture, to maintain the pH of 7.4, and was regularly replaced to prevent glucose depletion. After a short equilibration period of 30 min, the segments were gradually stretched (50 or 25 μm increments) to stresses above 13.3 kPa (100 mm Hg), as described earlier (Angus and Wright, 2000). After this passive stretch protocol, the segments were set at the internal circumference according to the 13.3 kPa stress (normalization factor = 0.9) (Slezák et al., 2010). The internal circumference of the different arteries was calculated as [(2 × Δμm stretch) × (4r) × (2rπ)] with r, the radius of the wire (20 μm). Then, all segments were re-set to zero force in order to measure active force (Van Hove et al., 2009). High K^+^-solutions were prepared by replacing NaCl with equimolar KCl. To avoid any vasomotor interference due to prostanoids, 10 μM indomethacin (Federa, Belgium) was present in the KR solution during all experiments.

### Isometric Organ Bath

Next, the thoracic aorta was carefully removed and stripped of adherent tissue. Starting at the diaphragm, the aorta was cut in 6 segments of 2 mm width using a calibrated stereomicroscope. Starting from the aortic arch, thoracic aorta (TA) segments TA2 to TA5 were immersed in KR solution, aerated with a 95% O_2_/5% CO_2_ gas mixture. Isometric contractions and relaxations were measured on segments TA4-TA5 by means of a Statham UC2 force transducer (Gould, United States). The aortic segments were stretched and stabilized to a preload of 25 mN. VSMCs were stimulated using the α_1_-adrenergic agonist phenylephrine (PE, 2 × 10^-6^ M) (Sigma-Aldrich, Belgium) and NΩ-nitro-l-arginine methyl ester (L-NAME, 3 × 10^-4^ M) (Sigma-Aldrich, Belgium) was used to inhibit the endothelial nitric oxide synthase (eNOS).

### Rodent Oscillatory Tension Set-Up to Study Arterial Compliance

The Rodent Oscillatory Tension Set-up to study Arterial Compliance (ROTSAC) is an in house developed organ bath that allows the isobaric assessment of biomechanical parameters of isolated mouse aortic segments that are cyclically stretched at physiological frequency (10 Hz). The aortic segments were mounted between two parallel wire hooks in 8 mL organ baths. As described previously (Leloup et al., 2016), the upper hook was connected to the aluminum lever of the force–length transducer. This lever was connected to a coil suspended in a strong field of a permanent magnet. The system was controlled by a current source. When current was passed through this coil, a force was developed. The displacement of the lever was measured by means of a photo-electric system. The force-length transducer was connected to a data acquisition system (Powerlab 8/30 and LabChart 7, ADInstruments) and acquisition speed was set at 1 kHz. To estimate the transmural pressure that would exist in the equilibrated vessel segment with the given distension force and dimensions, the Laplace relationship was used:

$$P=\frac{F}{l.D}$$

with F the force, l the length and D the diameter of the vessel segment. Force was measured directly by the transducer. The diameter of the vessel segment at a given preload was derived from the displacement of the upper hook, being directly proportional to the inner circumference:

$$D=\frac{2g}{\pi }$$

with g the outer distance between the hooks (to approximate the inner circumference of the vessel segment). Before each experiment, diameter and length were determined at three different preloads (20, 40, and 60 mN) using a stereomicroscope and calibrated image software. To correct for the slight decrease in vessel length with increasing diameter, the average segment length per cycle (100 ms) was derived from the diameter-length relationship using basic linear regression.

The preloads were adjusted until the diastolic and systolic pressure corresponded to 80–120 mmHg. Compliance (C) was calculated as follows:

$$C=\frac{\Delta D}{\Delta P}$$

with ΔD, the difference between systolic and diastolic diameter and ΔP, the pressure difference (±40 mmHg in the present study). The Peterson modulus of elasticity (Ep) is a frequently used, vessel size-independent measure of arterial stiffness (Gosling and Budge, 2003) and was calculated as follows:

$$Ep=D_{0}\cdot \frac{\Delta P}{\Delta D}$$

with D_0_, the diastolic diameter. During all experiments, the segments were periodically stretched directly after mounting them in the organ bath with a frequency of 10 Hz to mimic the physiological heart rate in mice (600 bpm). Every experimental protocol was performed on 2 segments (TA2-TA3) from a control and AngII-treated animal in parallel, thus using 4 organ baths in total. VSMCs were stimulated using PE (3 × 10^-8^ or 2 × 10^-6^ M) and L-NAME (3 × 10^-4^ M) was added to every second organ bath at the beginning of each experiment to inhibit NO production by eNOS. Diethylamine NONOate (DEANO, 2 × 10^-6^ M) was used to maximally relax VSMCs by activating soluble guanylate cyclase (sGC), the endogenous target of NO. The stiffness measurements were done at steady-state contractions, 30 min after the addition of PE or 5 min after the addition of DEANO. The protocol was the same for both segments (with and without L-NAME) and consisted of a baseline measurement in KR solution, two consecutive measurements in the presence of 3 × 10^-8^ M PE and 2 × 10^-6^ M PE, respectively. After a new measurement in fresh KR solution, DEANO was added to a final concentration of 2 × 10^-6^ M to relax all VSMCs.

### Statistics

All results are expressed as mean ± SD with n representing the number of mice, and analyzed using Prism 6.0 (GraphPad Software, La Jolla, CA, United States). When testing the treatment factor in combination with other factors (e.g., level of VSMC stimulation), a two-way ANOVA was used. To correct for multiple comparisons, a Bonferroni correction was applied. When comparing a single parameter between the treated and control group, a Student *t*-test was used. A non-parametric Mann–Whitney test was used when an *F*-test of equality of variances revealed significantly different variances. Paired testing was performed when possible. All statistical tests are mentioned in the figure legends. A 5% level of significance was selected.

## Results

### *In Vivo* Measurements

**FIGURE 1:**
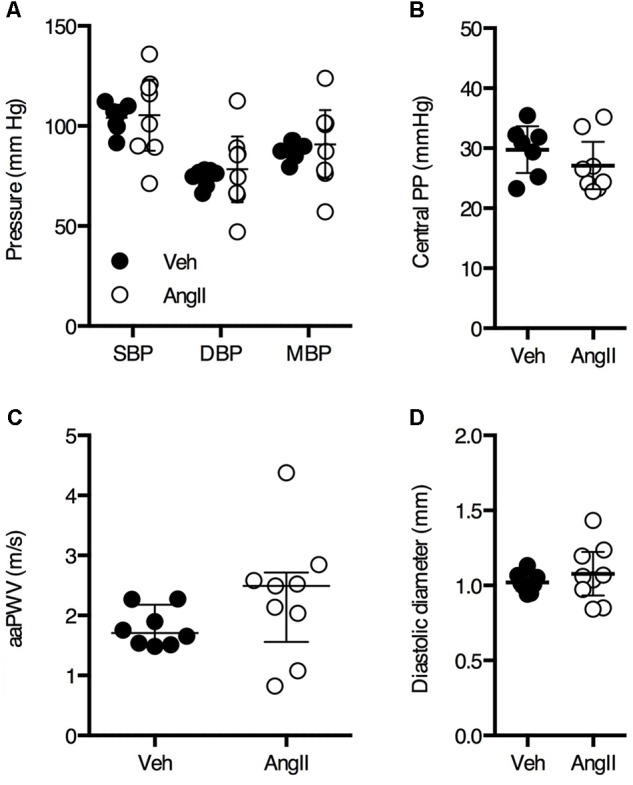
Invasive central blood pressure **(A,B)** measurements on anesthetized animals revealed no significant difference between vehicle-treated (Veh) and AngII-treated (AngII) mice (*n* = 7–9/group). Non-invasive ultrasound-based assessment of abdominal aorta pulse wave velocity (aaPWV) revealed a small but non-significant increase in aaPWV **(C)**. Abdominal aorta diastolic diameter assessed using ultrasound was not significantly different between both groups **(D)**. Unpaired Student’s *t*-test **(A,B,D)** or Mann–Whitney test **(C)**. Line and error bars represent mean and 95% confidence intervals **(A,B,D)** or median and interquartile range **(C)**, respectively.

After 1 week of AngII treatment, body weight was not significantly different between vehicle (32.6 ± 1.3 g, *n* = 9) and AngII-treated animals (32.3 ± 1.6 g, *n* = 10). Invasive pressure recordings were acquired from the aortic arch to assess central blood pressure. Surprisingly, blood pressure parameters were not significantly affected by 1 week of AngII-treatment (**Figures 1A,B**).

PWV is the most important *in vivo* parameter of arterial stiffness. There was a small but non-significant increase in aaPWV in AngII-treated mice as compared to controls (**Figure 1C**). Diastolic diameter of the abdominal aorta as measured by ultrasound was not significantly different between both groups (**Figure 1D**).

### Rodent Oscillatory Tension Set-Up to Study Arterial Compliance

**FIGURE 2:**
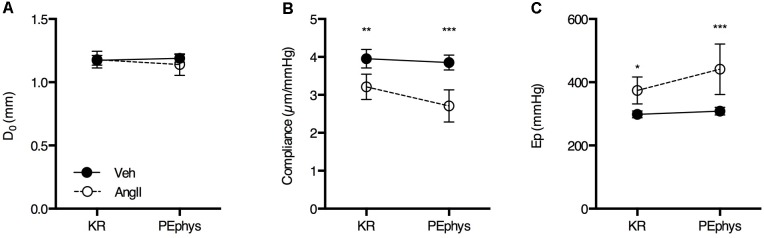
Basal diastolic diameter **(A)**, compliance **(B)**, and Ep **(C)** in KR and in the presence of 3 × 10^-8^ M PE (PEphys) to mimic the physiological basal α_1_-adrenergic stimulation by circulating catecholamines (*n* = 10/group). Repeated measures two-way ANOVA with Bonferroni *post hoc* test for multiple comparisons. The interaction was significant for diameter (*p* < 0.01), compliance (*p* < 0.001) and Ep (*p* < 0.001). ^∗^, ^∗∗^, ^∗∗∗^*p* < 0.05, 0.01, 0.001 vs. vehicle. Line and error bars represent mean and 95% confidence intervals.

In basal, unstimulated conditions (KR), isobaric diastolic diameter (80 mmHg) was not significantly different in AngII-treated mice (1.18 ± 0.09 mm, *n* = 10) as compared to controls (1.17 ± 0.05 mm, *n* = 9) (**Figure 2A**). Compliance was ∼20% lower in AngII-treated mice (3.21 ± 0.47 μm/mmHg, *n* = 10) (**Figure 2B**) as compared to controls (3.95 ± 0.32 μm/mmHg, *n* = 9) and Ep, a vessel size-independent measure of arterial stiffness, was ∼25% higher in AngII-treated mice (374 ± 60 mmHg, *n* = 10) as compared to controls (298 ± 14 mmHg, *n* = 9) (**Figure 2C**). To mimic basal α_1_-adrenergic stimulation by circulating catecholamines, a low dose of PE (3 × 10^-8^ M) was added to the organ bath. This affected diameter (**Figure 2A**), compliance (**Figure 2B**) and Ep (**Figure 2C**) significantly more in AngII-treated animals (interaction *p* < 0.01, *p* < 0.001, and *p* < 0.001, respectively) as compared to vehicle-treated mice, increasing the differences between both groups even further.

**FIGURE 3:**
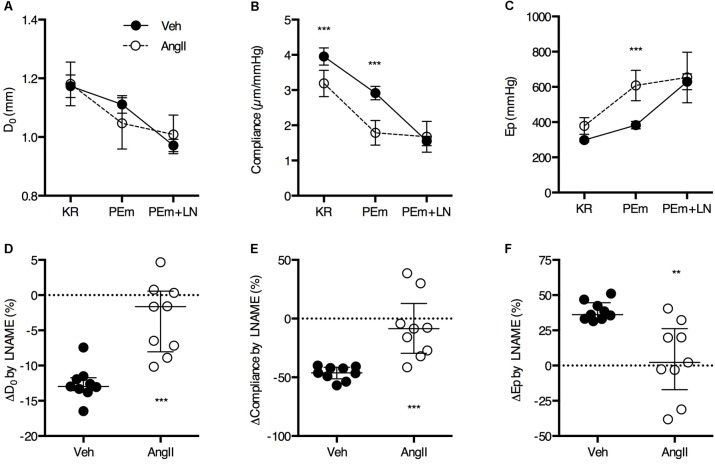
Basal NO bioavailability. Addition of 300 μM L-NAME (LN) to a maximally stimulating concentration of PE (2 μM, PEm) allows to estimate the amount of basal NO that suppresses the PEm contraction in the absence of LN **(A–C)**. The effect of basal NO blockade (i.e., LN effect) relative to the magnitude of the PEm effect was almost zero in AngII-treated mice (*n* = 10) and significantly smaller as compared to vehicle-treated mice (*n* = 9) **(D–F)**. Two-way ANOVA with Bonferroni *post hoc* test for multiple comparisons, there was a significant interaction between the treatment and VSMC contraction factor for D_0_ (*P* > 0.001), compliance (*P* < 0.001) and Ep (*P* < 0.05) **(A–C)**. Mann–Whitney test **(D–F)** ^∗^, ^∗∗^, ^∗∗∗^*P* < 0.05, *P* < 0.01, *P* < 0.001 vs. vehicle. Line and error bars represent mean and 95% confidence intervals **(A–C)** or median and interquartile range **(D–F)**, respectively.

Basal NO suppresses approximately 12% of the isobaric diameter contraction in vehicle-treated animals and only ∼4% in the AngII-treated animals (**Figures 3A,D**). In the vehicle-treated animals, this resulted in a ∼50% suppression of the reduction in compliance (**Figures 3B,E**) and a ∼40% increase in Ep (**Figures 3C,F**) by PE. The effects of L-NAME on isobaric diameter, compliance or Ep are virtually absent in the AngII-treated animals, indicating severely compromised basal NO bioavailability or efficacy (**Figures 3A–F**).

**FIGURE 4:**
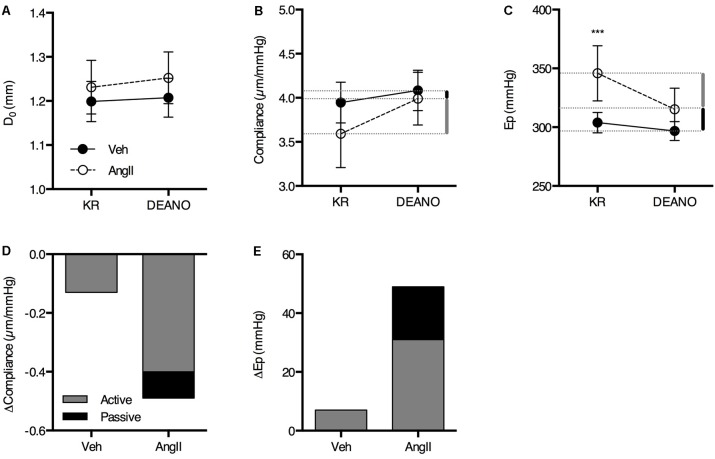
The contribution of active and passive factors in the increased aortic stiffness in AngII-treated mice. The effects of 2 μM DEANO on D_0_ **(A)**, compliance **(B),** and Ep **(C)** are significantly different in AngII-treated mice (*n* = 8) as compared to controls (*n* = 8). Diameter, compliance and Ep are not significantly different in fully relaxed aortic segments **(A–C)**. The contribution of active and passive factors to the increased aortic stiffness parameters in AngII-treated animals is indicated with the gray and black bars, respectively, in **(B,D)**, and **(C,E)**. Repeated measures two-way ANOVA with Bonferroni *post hoc* test for multiple comparisons. There was a significant interaction between the treatment and DEANO factor for diameter (*p* < 0.05), compliance (*p* < 0.01), and Ep (*p* < 0.01). ^∗^, ^∗∗^, ^∗∗∗^*P* < 0.05, *P* > 0.01, *P* > 0.001 vs. vehicle. Line and error bars represent mean and 95% confidence intervals, respectively.

The large effects of L-NAME suggest low basal NO bioavailability and, hence, increased VSMC tone. We previously showed that increased VSMC tone ‘actively’ affects aortic biomechanics in the healthy mouse aorta (Leloup et al., 2016). To assess the amount of structural stiffness (i.e., passive alterations of the aortic wall structure independent from VSMC or EC reactivity), the aortic segments were maximally relaxed using excess exogenous NO (2 μM DEANO). Interestingly, the effects of VSMC relaxation using DEANO on isobaric diameter, compliance and Ep were clearly elevated in the AngII-treated animals (**Figures 4A–C**). DEANO dose-response studies in the isometric organ bath revealed no increase in DEANO sensitivity or maximal relaxation in AngII-treated animals (data not shown), indicating that increased basal VSMC tone rather than increased DEANO efficacy is responsible for these effects. Removal of basal VSMC tone using DEANO also allowed estimation of the contribution of VSMC tone-dependent (‘active’) and VSMC tone-independent (‘passive’) factors to the isobaric biomechanics of the aortic wall (**Figures 4D,E**). Active stiffness (**Figure 4**, gray bars) was defined as the part of the stiffness difference with controls that could be removed through VSMC relaxation, while passive stiffness (**Figure 4**, black bars) was defined as the stiffness difference that remained between both groups when VSMCs were fully relaxed. These data clearly show that, in controls, the basal tone of VSMCs that contribute to the compliance or Ep of the aortic wall is low and baseline compliance is near-maximal, i.e., removal of basal VSMC tone with DEANO increases aortic wall compliance only slightly. However, in the AngII-treated mice, removal of VSMC tone resulted in a much larger decrease in average stiffness while only a minor part of the stiffness difference with controls remained after relaxation. This indicates that the major contributor of aortic stiffness in these animals was the increase of basal VSMC tone rather than passive aortic wall remodeling.

### Vascular Reactivity

**FIGURE 5:**
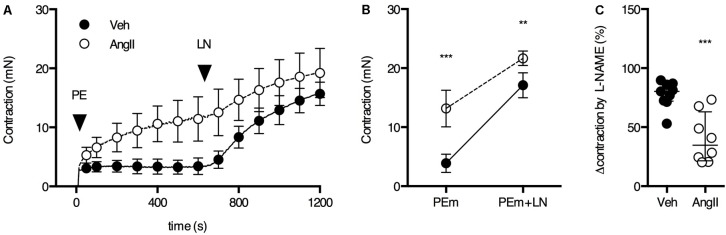
Basal NO in the aorta. **(A)** Maximal PE-induced contraction (2 μM PE, PEm) in the absence and presence of 300 μM L-NAME (LN). **(B)** The contraction was significantly larger in AngII-treated mice (*n* = 8–9) versus controls (*n* = 9) and this difference decreased when basal NO release was blocked (interaction *P* < 0.05). **(C)** The suppression of the contraction was significantly smaller in AngII-treated mice, indicating reduced basal NO efficacy. Two-way ANOVA with Bonferroni *post hoc* test for multiple comparisons. There was a significant interaction between the treatment and VSMC contraction factor (*P* < 0.05) **(B)**. Mann–Whitney test **(C)**. ^∗^, ^∗∗^, ^∗∗∗^ *P* < 0.05, *P* < 0.01, *P* < 0.001 vs. vehicle. Line and error bars represent mean and 95% confidence intervals **(A,B)** and median and interquartile range **(C)**, respectively.

The ROTSAC results indicated reduced basal NO efficacy in AngII-treated mice, leading to increased basal VSMC tone and, hence, increased isobaric stiffness. In order to confirm this, aortic segments were mounted in the traditional organ bath and basal NO release was determined by measuring the maximal PE contraction before and after the addition of 300 μM L-NAME to inhibit eNOS and basal release of NO (van Langen et al., 2012; Leloup et al., 2015). In the absence of L-NAME (first 600 s), the contraction induced by 2 μM PE was significantly larger in aortic segments of AngII-treated mice as compared to vehicle-treated animals (**Figures 5A,B**). In both groups, the PE-induced contraction increased significantly after eNOS inhibition with L-NAME. The response to L-NAME corresponds to the functional amount of basal NO and was signficiantly larger in vehicle-treated animals (**Figure 5B**). In AngII-treated mice, basal NO efficacy was severely compromised (**Figure 5C**), confirming the ROTSAC data showing increased aortic wall stiffness due to the impaired capacity of the endothelium to suppress the contractile effect of α_1_-adrenergic stimulation with PE (**Figure 3**).

**FIGURE 6:**
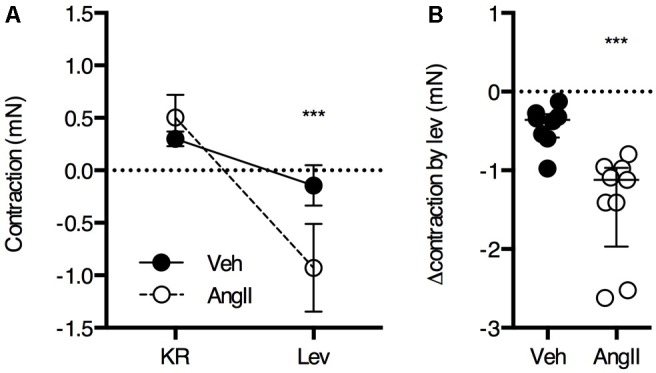
Basal Ca^2+^ influx was estimated from the reduction in isometric preload upon membrane hyperpolarization with levcromakalim (*n* = 8–9/group). Repeated measures two-way ANOVA with Bonferroni *post hoc* test for multiple comparisons, the interaction between the treatment and levcromakalim factor was significant (*P* < 0.001) **(A)**. Mann–Whitney test **(B)** ^∗∗∗^*P* < 0.001 vs. vehicle. Lines and error bars represent mean and 95% confidence intervals **(A)** or median and interquartile range **(B)**, respectively.

The ROTSAC data revealed a high contribution of basal VSMC tone to the increased isobaric stiffness in AngII-treated mice. To confirm increased basal Ca^2+^ influx in the aortic VSMCs of the AngII-treated mice, the drop in isometric preload upon the addition of 1 μM levcromakalim was measured (**Figure 6**). As expected, the effect of levcromakalim was small in controls (∼0.5 mN), confirming previous observations that, in the healthy, unstimulated aorta, the contribution of VSMC tone to isobaric stiffness is low. In AngII-treated mice, the effect of VSMC hyperpolarization with levcromakalim was significantly larger (∼1.5 mN) as compared to controls, confirming the ROTSAC data showing increased contribution of VSMC tone to baseline aortic wall stiffness (**Figure 4**).

**FIGURE 7:**
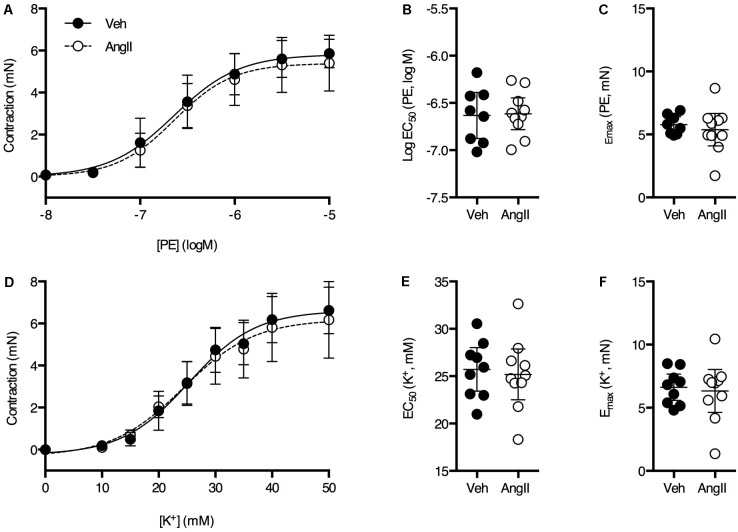
PE dose response **(A)** and K^+^ dose-response **(D)** curves were similar in both groups (*n* = 8–10/group). Fitted EC_50_ and E_max_ values for PE **(B,C)** and K^+^ **(E,F)** were not significantly different between vehicle-treated or AngII-treated mice. Unpaired Student’s *t*-test. Lines and error bars represent mean and 95% confidence intervals, respectively.

**FIGURE 8:**
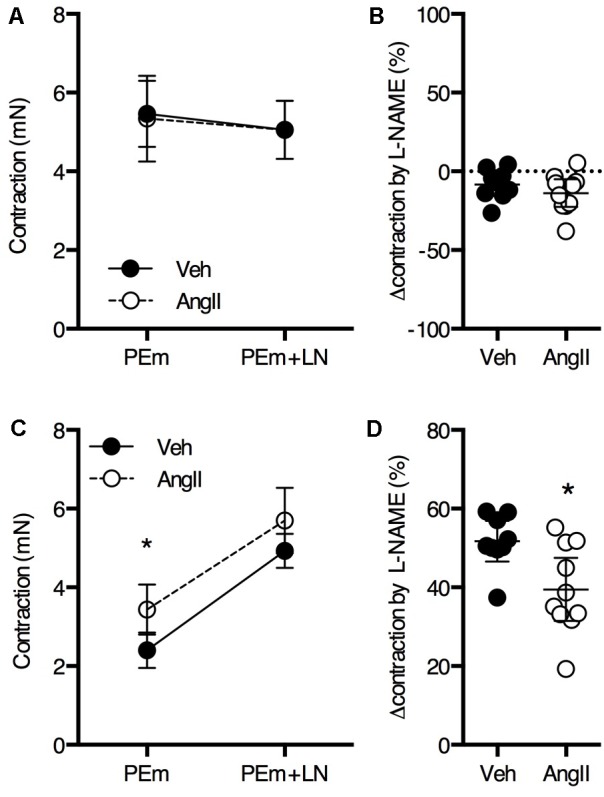
Basal NO in the femoral **(A,B)** and carotid **(C,D)** artery. The maximal PE-induced contraction (2 μM PE, PEm) of femoral artery segments (*n* = 9–10) was similar in the absence and presence of 300 μM L-NAME (LN) **(A)**. The change in maximal contraction upon addition of L-NAME was not significantly different between AngII-treated and vehicle-treated animals **(B)**. In carotid artery segments (*n* = 9–10), maximal PE-induced contraction increased upon addition of L-NAME (*P* < 0.05 for treatment factor) **(C)**. The increase in contraction upon blocking basal NO using L-NAME was significantly larger for AngII-treated animals **(D)**. Repeated measures two-way ANOVA with Bonferroni *post hoc* test for multiple comparisons **(A,C)** and unpaired Student’s *t*-test **(B,D)**. ^∗^*P* < 0.05 vs. vehicle. Line and error bars represent mean and 95% confidence intervals, respectively.

Overall, thoracic aorta biomechanics and reactivity were shown to be severely affected by a 1-week AngII treatment. Because we previously showed that a 4-week AngII treatment differentially affected large and small arteries, vascular reactivity analysis of femoral artery segments was performed. No significant effects of the AngII treatment on PE (**Figures 7A–C**) and K^+^ (**Figures 7D–F**) sensitivity and efficacy on the femoral artery isometric contraction could be observed. As demonstrated earlier (Leloup et al., 2015), basal NO was absent in the femoral artery and AngII-treatment did not affect this (**Figures 8A,B**). Myography was also performed on carotid artery segments and the effect of the AngII-treatment on basal NO efficacy showed a similar trend as compared to the aorta (**Figures 8C,D**), indicating that, in the present study, short term AngII-treatment specifically affected the *ex vivo* reactivity and biomechanics of the large, elastic arteries without affecting *ex vivo* femoral artery reactivity and *in vivo*, anesthetized central blood pressure parameters and aaPWV.

## Discussion

AngII-treatment is a well-known and commonly used method to induce arterial hypertension in mice. The present study aimed to investigate the effect of a short-term AngII treatment on the active biomechanical properties of the large arteries. Remarkably, no difference in central blood pressure was found. A small but non-significant increase in *in vivo* abdominal aorta stiffness, as measured by aaPWV, was observed. Isobaric *ex vivo* stiffness was increased in AngII-treated mice aortic segments. This was – at least partly – caused by a reduced basal NO efficacy and an increased basal VSMC tone in AngII-treated animals. Indeed, elimination of all basal VSMC Ca^2+^ influx reduced stiffness and isometric preload significantly more in AngII-treated mice. Vascular reactivity studies of aortic segments confirmed the alterations in active biomechanical properties as seen in the ROTSAC set-up. The chronic, irriversible effects of AngII treatment on smaller vessels were assessed by analyzing vascular reactivity of femoral artery segments. Interestingly, no effects of AngII could be detected, suggesting that in the present study, the vascular defects caused by chronic AngII infusion were selective for the elastic arteries.

### Blood Pressure and PWV

In the present study, body weight and anesthetized central blood pressure were unaffected by 1-week AngII infusion. This is in contrast to our previous study where we demonstrated that a 4-week AngII treatment resulted in hypertension (Fransen et al., 2016). The lack of a significant increase in BP after 1 week AngII infusion (1000 ng.kg^-1^.min^-1^) in adult, male C57Bl/6J mice has been reported before (Young Park et al., 2012). However, the vast majority of studies on adult, male C57Bl/6J mice report a significant ∼10–60% increase in SBP after 1-week of treatment with AngII (Young et al., 2012; Majeed et al., 2014; Glass et al., 2015; Xie et al., 2015). A study where 694 ng.kg^-1^.min^-1^ [Val^5^]-AngII was administered using osmotic minipumps showed a 36% increase in concious SBP after 5 days. Interestingly, the treatment did not affect PWV in ketamine-xylazine anesthetized mice (Fry et al., 2015). PWV is strongly BP-dependent (Shadwick, 1999) so one would expect increased PWV in [Val^5^]-AngII-treated mice, even if aortic stiffness was the same. This suggests that anesthesia lowered central BP more in the [Val^5^]-AngII-treated animals and this might explain why central BP measurements, aaPWV and diastolic diameter of isoflurane-anesthetized animals were not significantly affected in the present study.

Unlike the lack of a significant effect of AngII treatment on central, anesthetized BP, 1-week AngII infusion strongly affected vascular reactivity and biomechanics of thoracic aorta segments.

### Basal NO and VSMC Tone

We described earlier that the isolated large, elastic arteries, but not the muscular arteries, produce large amounts of basal NO (Leloup et al., 2015) and that basal NO plays a crucial role in determining the effects of α_1_-adrenergic stimulation on the isobaric biomechanical properties of the healthy mouse aortic wall (Leloup et al., 2016). In the present study we observed a significant effect of AngII treatment on the efficacy of basal NO in the aorta, resulting in an increase in isobaric stiffness at moderate (physiological) or maximal α_1_-adrenergic stimulation. This was also indicated by the increased response of the isometric contraction on PE. Both the isobaric stiffness and the isometric contraction differences diminished upon the addition of the eNOS blocker L-NAME.

To assess whether the basal increase in isobaric Ep in the AngII-treated mouse aorta could be reversed by compensating for the reduced basal NO efficacy, exogeneous NO was added to the organ bath to maximally stimulate sGC, and to remove basal VSMC tone. There was indeed a significant interaction between the effect of DEANO on isobaric compliance and Ep and the AngII treatment, indicating that the lack of basal NO efficacy and/or increased VSMC tone in the treated group are indeed responsible for the observed increase in isobaric stiffness. The addition of DEANO could eliminate most of the isobaric stiffness difference with controls. The remaining difference with fully relaxed VSMC tone was small, indicating that – in this mouse model – the majority of the AngII-mediated stiffening was due to active, VSMC-tone-mediated changes rather than structural remodeling of the aortic wall. Endothelial dysfunction, typically characterized by a reduced bioavailability of NO, has been observed previously in experimental models of vascular disease, including the diet-induced obesity mouse model (Weisbrod et al., 2013), pre-atherosclerotic apoE^-/-^ mice (Fransen et al., 2008) and rodent models of hypertension (Küng and Lüscher, 1995) and is a common and well recognized early feature of vascular aging in humans (Kaess et al., 2012). As mentioned earlier, we previously demonstrated a significant contribution of VSMC tone and especially basal NO to isobaric stiffness parameters of the healthy mouse aorta *ex vivo* (Leloup et al., 2016). It is, however, the first time that dysfunctional active vessel wall components are demonstrated to be the major contributors of increased isobaric aortic stiffness in an animal model of vascular disease.

We confirmed the increase in VSMC tone in the isometric organ bath using levcromakalim instead of DEANO. Levcromakalim opens ATP-dependent K^+^ channels and hyperpolarizes the resting membrane potential of VSMC to the K^+^-equilibrium potential, thereby blocking Ca^2+^ influx via voltage-gated Ca^2+^ channels (Knot and Nelson, 1998; Fransen et al., 2012). Indeed, the fall in isometric preload upon addition of levcromakalim was significantly larger in AngII-treated mouse aortic segments, confirming the observations from the ROTSAC set-up that basal VSMC tone is increased after 1 week of AngII treatment.

The present study reveals a remarkable site-specificity of the non-acute effects of AngII-infusion on the vasculature. Even though aorta and carotid artery stiffness and reactivity were affected, *in vivo* aaPWV and femoral artery reactivity remained unchanged. This might seem remarkable as we previously showed that a 4-week AngII treatment increased the maximal contraction by 50 mM K^+^ and desensitized the isometric contractions to depolarization, as indicated by a rightward shift of the K^+^ dose-response curve, in femoral artery segments of the AngII-treated animals (Fransen et al., 2016). However, the present study suggests that the effects of AngII in the large arteries occur predominantly via altered basal NO signaling. Indeed, AngII is known to affect NO signaling (e.g., via eNOS uncoupling and increased oxidative stress) (Nakashima et al., 2006). Because muscular arteries, such as the femoral artery, do not produce basal NO in the organ bath (Leloup et al., 2015), any effects of the AngII treatment on basal NO signaling will not be reflected in the PE or K^+^ response in femoral artery segments or on the regional aaPWV of the relatively muscular abdominal aorta. Therefore we speculate that the non-acute effects of AngII-infusion specifically affect basal NO signaling and, hence, large artery function, while muscular artery function is affected only after prolonged AngII infusion. Further research is needed to confirm the time- and site-specificity of the effects of AngII-infusion on arterial reactivity and biomechanics.

To summarize, in this paper the effects of short-term (1 week) AngII treatment on the functional and biomechanical properties of isolated arterial segments of the mouse were analyzed. Unlike the lack of significant effects on *ex vivo* femoral artery reactivity, anesthetized central BP parameters and aaPWV, AngII treatment strongly affected large artery function and biomechanics. Isobaric aortic stiffness was increased and, interestingly, a major part of the increase could be attributed to reduced basal NO efficacy and increased VSMC tone. This is, to our knowledge, the first time that dysfunctional active vessel wall components are shown to be the major cause of increased aortic stiffness in a mouse model of arterial disease. Even though further research on the temporal relationship between active vessel wall component dysfunction, arterial stiffness and hypertension in this specific mouse model is needed to confirm the remarkable lack of significant *in vivo* effects, several observations strongly indicate that the active components of the large arteries can contribute to the pathogenesis of large artery stiffening and are potentially promising targets to treat early vascular aging to prevent the associated CV complications.

## Author Contributions

AL, SDM, CVH, LD, ZV, and PF conceived and designed the experiments. Acquisition, analysis and interpretation of data were carried out by AL, SDM, CVH, and PF in the laboratories of VS, GDK, and PF. The manuscript was drafted and critically revised by AL, SDM, CVH, LD, ZV, VS, GDK, and PF. All authors have approved the final version of the manuscript and agree to be accountable for all aspects of the work.

## Conflict of Interest Statement

The authors declare that the research was conducted in the absence of any commercial or financial relationships that could be construed as a potential conflict of interest. The reviewer CA and handling Editor declared their shared affiliation.
